# Evaluating the sensitivity and specificity of Determine™ HIV-1/2 rapid test using a 0.01M phosphate-buffered saline produced at the Medical Research Council Unit The Gambia for the diagnosis of HIV

**DOI:** 10.1093/trstmh/trad071

**Published:** 2023-10-11

**Authors:** Anna Boté-Casamitjana, Tisbeh Faye-Joof, Ousman Bah, Sira Jallow, Alagie Camara, Olimatou Jallow, Nuredin Mohammed, Karen Forrest, Behzad Nadjm

**Affiliations:** Medical Research Council Unit The Gambia, London School of Hygiene and Tropical Medicine, Atlantic Boulevard, The Gambia; Medical Research Council Unit The Gambia, London School of Hygiene and Tropical Medicine, Atlantic Boulevard, The Gambia; Medical Research Council Unit The Gambia, London School of Hygiene and Tropical Medicine, Atlantic Boulevard, The Gambia; Medical Research Council Unit The Gambia, London School of Hygiene and Tropical Medicine, Atlantic Boulevard, The Gambia; Medical Research Council Unit The Gambia, London School of Hygiene and Tropical Medicine, Atlantic Boulevard, The Gambia; Medical Research Council Unit The Gambia, London School of Hygiene and Tropical Medicine, Atlantic Boulevard, The Gambia; Medical Research Council Unit The Gambia, London School of Hygiene and Tropical Medicine, Atlantic Boulevard, The Gambia; Medical Research Council Unit The Gambia, London School of Hygiene and Tropical Medicine, Atlantic Boulevard, The Gambia; Medical Research Council Unit The Gambia, London School of Hygiene and Tropical Medicine, Atlantic Boulevard, The Gambia

**Keywords:** Determine, HIV, phosphate buffer saline, POCTs, rapid test, stockouts

## Abstract

**Background:**

Human immunodeficiency virus (HIV) rapid diagnostic tests (RDTs) are widely used. However, buffer stockouts commonly lead to utilising non-approved liquids, resulting in errors. Our aim was to evaluate the diagnostic accuracy of an alternative buffer.

**Methods:**

Paired Determine HIV-1/2 rapid tests with commercial buffer and locally produced 0.01M phosphate-buffered saline (PBS) were performed on consecutive consenting individuals requiring HIV testing. Serum samples were sent for confirmation through the local gold-standard algorithm (Murex HIV Ag/Ab, Hexagon HIV with/without Geenius HIV 1/2). Test accuracy, κ and exact McNemar's test were also carried out.

**Results:**

Of 167 participants, 137 had confirmatory testing. The sensitivity of the Determine HIV-1/2 test using PBS compared with the gold standard was 100% (95% confidence interval [CI] 90.5 to 100) with a specificity of 98% (95% CI 92.9 to 99.8). The κ value was 0.94 compared with the gold standard and 0.92 compared with the Determine HIV-1/2 test using the commercial buffer. McNemar's test showed no evidence of differing sensitivities. Due to operational constraints, the study included 37 of the 49 positive cases as determined by the sample size calculation, resulting in an attained power of 80% instead of the intended 90%.

**Conclusions:**

These results suggest that 0.01M PBS is an alternative solution for Determine HIV-1/2 when buffer stockouts occur.

## Introduction

Human immunodeficiency virus (HIV) remains a significant public health problem worldwide. Despite almost reaching Joint United Nations Programme on HIV/AIDS (UNAIDS) 90-90-90 global targets at the end of 2020,^1^ many people worldwide do not know their status or receive any treatment, gaps that are even larger in some regions or subpopulations. The Gambia's overall adult HIV prevalence is 1.8% (95% confidence interval [CI] 1.4 to 2.3)^2^ and among the 27 000 (95% CI 21 000 to 35 000) people living with HIV (PLHIV), just 51% (95% CI 40 to 66) are aware of their status.^1^

As the HIV epidemic evolves and testing services become increasingly available, positive results among all tests are expected to decline. Hence the World Health Organization (WHO) recommends that countries with a national prevalence of <5% use three sequential tests to make a correct diagnosis. This should include rapid diagnostic tests (RDTs) or enzyme immunoassays; more complex techniques should be avoided.^3^

RDTs are cheap, widely available and easy to perform. Trained healthcare workers can perform these, laboratory facilities or complicated infrastructure are not required and reagents can be stored at 0–30°C.^4–6^ RDTs reduce the turnaround time and offer same-day results, increasing the knowledge of HIV status and the opportunity to provide early counselling and treatment.^7–10^

Nonetheless, test accuracy should be individually validated nationally before implementing local algorithms. Several studies in different low- and middle-income countries (LMICs) have reported test accuracy below the minimal WHO requirements.^11–14^ Explanations for these results include cross-reactivity with malaria,^15,16^ schistosomiasis^17^ or *Trypanosoma brucei* antibodies,^18^ insufficient personnel training or poor-quality performance.^14,19–21^ Quality issues, packaging problems and buffer stockouts are also common. In Nigeria, among 85 interviewed healthcare practitioners using malaria RDTs, 76.2% had run out of buffer, of whom 73% utilised alternative reagents.^22^ Moreover, empty buffer vials, erratic volumes and leaking vials have also been documented.^23,24^

The buffer is critical in maintaining an optimal pH and ionic strength. Alternative liquid solutions can decrease the lateral flow rate along the nitrocellulose strip or generate non-specific antibody–antigen complexes by modifying pH conditions, leading to errors.^22,25^

Evidence evaluating the use of substitute buffers is scarce. False positive and invalid results have been described when distilled or tap water or 0.9% saline is used to test individuals for malaria and coronavirus disease 2019 (COVID-19).^25,26^ In the HIV field, Kingston et al.^27^ reported that 0.9% saline use with the Determine HIV-1/2 rapid test had a sensitivity and specificity of 100% compared with the commercial buffer, but tap water produced 40% invalid results. Similarly, another study using the SD-Bioline HIV rapid test found that 0.85% normal saline detected 97.3% of all positive samples.^28^ However, these studies were done retrospectively, with unclear blinding strategies, not reflecting test field conditions and comparisons were made with non-standard tests.

0.01M phosphate-buffered saline (PBS), a water-based salt solution that is easy to prepare and widely used for biological purposes, was found to provide reliable results when used with the Determine HIV-1/2 rapid test in Mwanza, Tanzania.^29^

HIV misdiagnosis due to buffer shortages and poorly validated substitute reagents can have significant consequences, including individual and social costs. People incorrectly diagnosed as HIV positive undergo lifelong and potentially harmful treatments and experience significant legal, ethical and psychological consequences. Therefore, testing an alternative buffer solution is essential to better understand its performance compared with the manufacturer's buffer and gold standard.

Although the manufacturer's buffer is preferred, and there is no better solution for this complex problem than preventing buffer scarcity or stockouts, this project aimed to evaluate an alternative buffer solution that could be safely used when these issues arise. Specifically, this project aimed to evaluate the usefulness of a locally produced 0.01M PBS for the diagnosis of HIV using the Determine HIV-1/2 rapid test (Abbott Diagnostics Medical, Chiba, Japan). The hypothesis was that this has a sensitivity of 95% (±5%) compared with the local gold-standard algorithm.

The study's primary objective was to estimate the test parameters and accuracy of the Determine HIV-1/2 test using PBS following the Standards for Reporting of Diagnostic Accuracy Studies guidelines. The secondary objectives were to assess the agreement between PBS and commercial buffer, to evaluate the performance of the Determine HIV-1/2 test using the commercial buffer in the setting and its clinical utility and to generate relevant information on the implementation of point-of-care tests (POCTs) in the Medical Research Council Unit The Gambia (MRCG).

## Methods

The study was approved by the ethics committee at the London School of Hygiene and Tropical Medicine and The Gambia government/Medical Research Council (MRC) Joint Ethics Committee (reference 27251). All participants were given a study information letter and written consent was obtained. The project staff translated consent forms into local languages and witnesses ensured that the information provided was accurately conveyed to the patient.

### Study setting and population

The study was conducted between June and August 2022 at the MRCG, located in the western part of the country near the capital Banjul. The Clinical Services Department provides healthcare to staff members, study participants and the general public. The clinic is located in an urban area, but it serves individuals from all over the country, including those from rural settings. It comprises a 42-bed ward and an outpatient clinic. A clinical audit conducted in 2021 revealed that the local HIV prevalence was 18% (K. Forrest, personal communication).

### Study design

Patients consented to the HIV test, in line with national guidelines. They were then approached by trained research staff for informed consent in local languages. The primary inclusion criteria for the participants were all adults and children >18 months of age (due to early maternal antibodies transference during pregnancy, birth and breastfeeding). The only exclusion criterion was individuals with needlestick injuries.

At enrolment, all individuals had RDTs performed by trained personnel using the commercial buffer and one using PBS by the research team.

A total of 100 µl of whole blood were collected with a capillary tube to load commercial and PBS buffer strips simultaneously. If individuals had already been pricked to perform the commercial test, 50 µl of the remaining whole blood in the syringe collected for serology was used or an extra prick test was performed to obtain the 50-µl capillary blood sample. The RDT using the commercial buffer and that using PBS were kept separate from inoculation until after the results had been recorded.

PBS was added to the strip after 1 min, allowing the sample to flow along the membrane, and results were read visually after 20 min, away from the patients and the clinical team, to minimise observer bias and ensure an objective reading. The PBS RDTs were read by one member of the study team, who never revealed the results to the clinical staff or the patients. In addition, they were also blind to the commercial buffer performance and clinical details.

The interpretation and disclosure of the results from the commercial RDTs were conducted in accordance with the recommendations provided by the kit manufacturer and the standard operational procedures in place. To maintain the integrity and reliability of the samples and reagents, the expiration dates were recorded in the electronic system at the time of testing. Confidentiality was assured throughout the process and post-test counselling was provided in accordance with national guidelines.

Regardless of the results of the RDTs, clinicians were instructed to request serum samples through the electronic system to confirm the results using the local gold-standard algorithm. In the wards, the nurse responsible for patient care collected the sample once the request had been made. In the outpatient department, however, a dedicated phlebotomy room within the same facility was utilised for blood collection. Thus patients were specifically instructed to visit the phlebotomy room.

The serum samples were sent to the MRCG serology laboratory on the same day to confirm the results with the local gold-standard algorithm. This consisted of a Murex HIV Ag/Ab combination test (DiaSorin, Dartford, UK), performed to confirm the results of the RDT, and Hexagon HIV (HUMAN Gesellschaft für Biochemica und Diagnostica, Weisbaden, Germany), utilised as a second test to confirm infection and differentiate between HIV-1 and HIV-2 infection. If Hexagon detected dual infection, results were confirmed with Geenius HIV 1/2 (Bio-Rad, Marnes-la-Coquette, France). Laboratory technicians could not access clinical information or rapid test results. And thus were blinded to the previous rapid test results.

### PBS production

PBS was produced at the MRCG serology laboratory using PBS commercial tablets (Sigma-Aldrich, Darmstadt, Germany). One tablet was diluted for 10 min with 200 ml of deionised water in a sterilised 500 ml glass bottle producing 0.01M phosphate buffer, 0.0027M potassium chloride and 0.137M sodium chloride (pH 7.4, at 25°C). The buffer was then sterilised in an autoclave at 136°C for 45 min, stored at room temperature in the 500-ml glass bottle and kept in a safe space to avoid contamination. The buffer was then transferred into 5-ml cleaned dropper bottles for clinical use.

The pH was monitored before and after autoclaving, every time the buffer was transferred into dropper bottles and every 15 d until the end of the study. The pH was stable between 7.40 and 7.60.

The cost to produce the PBS solution was £0.0015 per test, while the price per test for the commercially purchased buffer was £0.062. It is important to note that this cost comparison does not consider autoclaving, deionised water and pH monitoring, which are necessary for the self-preparation process but do not apply to the commercially purchased buffer.

### Statistical methods

Statistical analysis was performed using STATA version 17 (StataCorp, College Station, TX, USA). Considering a sensitivity of 99.99% for the gold-standard algorithm and an estimated reduction of 5% using Determine HIV-1/2, 48 positive results were deemed necessary to accomplish statistical power (0.90 power).

**Figure 1.**
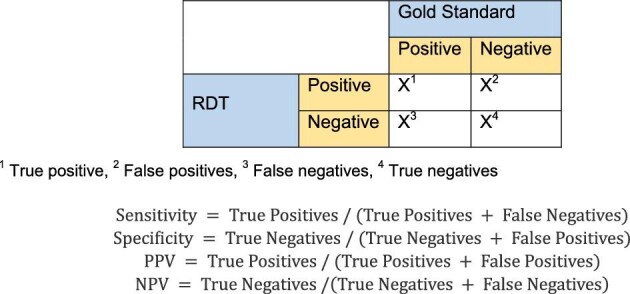
2×2 table to evaluate test accuracies and utilised formulas.

Sensitivity, specificity, positive predictive value (PPV), negative predictive value (NPV) and their 95% CIs were measured to evaluate the test accuracy and validity of Determine HIV-1/2 using PBS and commercial buffer (Figure 1). The κ coefficient was carried out to evaluate the agreement between tests. The exact McNemar's or Stuart–Maxwell test with an approximate χ^2^ distribution with k−1 degrees of freedom (df) was utilised to test the null hypothesis.

## Results

### Characteristics of the population

**Figure 2.**
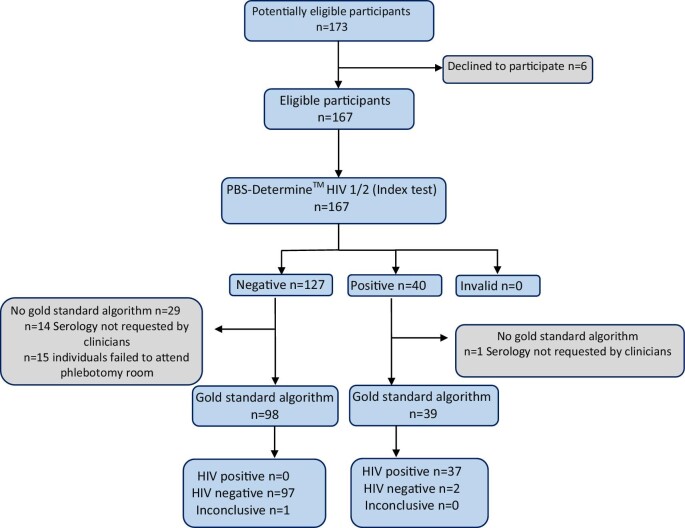
Study flow diagram evaluating the Determine HIV-1/2 test using 0.01M PBS compared with the local gold-standard algorithm or confirmatory test (Murex HIV Ag/Ab and Hexagon HIV).

A total of 173 individuals were screened for enrolment between 16 June and 31 August 2022. Of these, 167 (96.5%) agreed to participate in the study (Figure 2).

The characteristics of individuals are summarised in Table 1. The median age of the participants at enrolment was 35 y (interquartile range [IQR] 25). The age range was broad (18 months–76 years). Of the total, 13.8% (23/167) were <16 y of age and in 23 cases (13.8%) the tests were performed on asymptomatic mothers whose children were sick and had an indication for HIV testing but were <18 mon. A total of 47.9% (80/167) were females and, among adults (>16 y of age), 68.1% (98/144) were married. The most common ethnicity was Mandinka (42.5% [71/167]), followed by Fula (19.2% [32/167]) and Wolof (12.6% [21/167]).

**Table 1.** Characteristics of individuals enrolled in the study (N=167)

Characteristics	Values	Individuals, n (%)
Age (years), mean±SD	35.42±18.19	167
Sex	Female	80 (47.90)
	Male	87 (52.10)
Age group (years)	<16	23 (13.77)
	16–24	30 (17.96)
	25–34	32 (19.16)
	35–44	28 (16.77)
	45–54	29 (17.37)
	≥55	25 (14.97)
Place of testing	OPD	106 (63.47)
	Ward	61 (36.53)
Marital status (children <16 excluded) (6 missing values)	Married	98 (68.05)
	Single	22 (15.27)
	Widowed	11 (7.63)
	Divorced	4 (2.70)
	In a relationship	3 (2.08)
Ethnic group (8 missing values)	Mandinka	71 (42.51)
	Fula	32 (19.16)
	Wolof	21 (12.57)
	Jola	12 (7.19)
	Serahule	9 (5.39)
	Manjago	3 (1.80)
	Others	11 (6.59)

SD: standard deviation; OPD: outpatient department.

The patients’ clinical characteristics at presentation that led the medical team to request a test are presented in Table 2. Weight loss was present in 35.3% (59/167) and unexplained fever or chronic cough was seen in 22.8% (38/167). In 3.6% (6/167), the only reason for testing was for screening purposes.

**Table 2.** Clinical characteristics at presentation

Symptoms at testing	Values, n (%)
Weight loss	59 (35.33)
Cough	38 (22.75)
Unexplained fever	38 (22.75)
Skin rash	22 (13.17)
Chronic diarrhoea	18 (10.78)
Cytopenia	16 (9.58)
Other symptoms	14 (8.38)
Oral *Candida*	10 (5.99)
Hepatosplenomegaly	7 (4.19)
Lymphadenopathy	6 (3.59)
Screening purposes (asymptomatic)	6 (3.59)
Malnutrition/failure to thrive	5 (2.99)
Central nervous system symptoms/infection	3 (1.80)
Unexplained liver disease	7 (4.19)
Sexually transmitted infections	2 (1.20)
Polyneuropathy	1 (0.60)

Among all tested individuals, 3% (5/167) had been diagnosed with tuberculosis and the diagnosis was suspected in 31.8% (53/167). Ten individuals (6%) were tested following a positive hepatitis B serology result and two (1.2%) following a diagnosis of nephrotic syndrome. Lymphoma was suspected in 1.8% (3/167) of the cases.

The prevalence of HIV among individuals was 27.2% (37/136), considering only individuals with three consecutive positive results (RDT, Murex Ab/Ag or Hexagon) as confirmed diagnoses, as per the WHO guidelines. Most infections were caused by HIV-1. Only one case (2.7% [1/37]) was positive for HIV-2, resulting in a cohort prevalence of <1% (1/136).

### Test accuracy of Determine HIV-1/2 using PBS compared with the reference standard

Of the 167 Determine HIV-1/2 RDTs conducted using PBS, 127 results were negative, 40 were positive and no invalid results were observed. Among the tested individuals, 30 (18%) did not have a confirmatory test using the gold-standard algorithm. Notably, this lack of confirmation was more prevalent in individuals who initially tested negative on the POCT, where the gold standard did not confirm 22.8% (29/127) of the results. This situation occurred only once when the rapid test yielded a positive result (1/40). In 50% of cases (15/30), although the clinical team had requested serology and advised patients to visit the phlebotomy room for blood tests, the patients failed to attend. The remaining 50% of cases resulted from a lack of requests by the clinicians. Furthermore, this issue was more frequently observed in the outpatient department, where 23.6% (25/106) of the results did not have a follow-up confirmatory test, compared with only 8.2% (5/61) of tests conducted in the wards (Figure 2).

This resulted in 137 total matched pairs, but 1 was excluded from test accuracy analyses due to indeterminate results with the gold-standard algorithm or confirmatory results. Results are presented in a 2×2 table (Table 3). Thirty-seven subjects had the condition of interest according to the gold-standard diagnosis and 99 did not. The number of false positives was two and there were no false negative results.

**Table 3.** Performance of the Determine HIV-1/2 test using PBS compared with the gold-standard algorithm (Murex HIV Ag/Ab and Hexagon HIV)

		Gold standard (Murex HIV Ag/Ab and Hexagon HIV)		
	Result	Positive	Negative	Total
Determine HIV-1/2 with PBS	Positive	37	2	39
	Negative	0	97	97
Total		37	99	136

The sensitivity of the Determine HIV-1/2 test using PBS compared with the gold standard was 100% (95% CI 90.5 to 100) and the specificity was 98% (95% CI 92.9 to 99.8). The PPV was 94.9% (95% CI 82.7 to 99.4), or in other words, nearly 95% of individuals with a positive RDT result were correctly identified as having the disease. The NPV was 100% (95% CI 96.3 to 100).

The test agreement was high at 97.8%, with a κ of 0.94 (p<0.0001), and the Stuart–Maxwell test (df 2) resulted in a p-value of 0.22, indicating that there is no evidence to reject the null hypothesis in favour of the alternative that the marginal proportions are different.

### Determine HIV-1/2 using PBS compared with the commercial buffer

Rapid tests using PBS and commercial buffer were performed on 167 individuals. On one occasion, the result of the commercial Determine HIV-1/2 test was not recorded. Among the 166 paired results, there were 161 concordant results and five discordant. Thirty-eight subjects were identified as HIV positive with both tests (Table 4).

**Table 4.** Performance of the Determine HIV-1/2 test using PBS compared with the commercial buffer

		Commercial buffer		
	Result	Positive	Negative	Total
PBS	Positive	38	2	40
	Negative	3	123	126
Total		41	125	166

The κ value was 0.92 and the exact McNemar's test (df 1) demonstrated that test accuracies are equivalent between both tests (p=0.66).

### Test accuracy of Determine HIV-1/2 using commercial buffer compared with the reference standard

**Figure 3.**
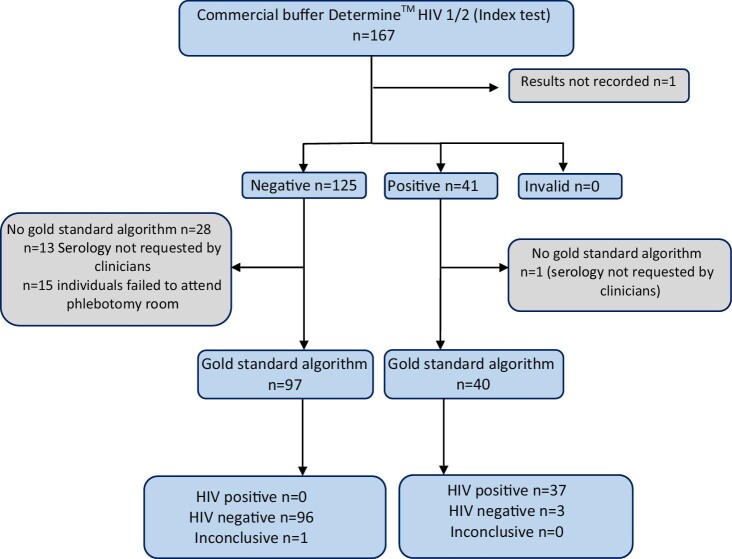
Study flow diagram evaluating the Determine HIV-1/2 test using commercial buffer compared with the local gold-standard algorithm or confirmatory test (Murex HIV Ag/Ab and Hexagon HIV).

A total of 167 individuals were tested using the Determine HIV-1/2 test with commercial buffer. The result for one individual was not documented. Of the 166 recorded results, 125 were negative, 41 were positive and there were no invalid results. Twenty-nine had no confirmatory test with the gold-standard algorithm, thus there were 137 matched pairs. One was excluded due to indeterminate results with the gold standard (Figure 3).

The RDT correctly identified all 37 individuals with a positive result with the gold-standard algorithm. Three of the 99 HIV-negative results were positive with the rapid test (false positives). These were all recorded by clinical staff as faint lines (Table 5).

**Table 5.** Performance of the Determine HIV-1/2 test using commercial buffer compared with the gold-standard algorithm

		Murex HIV Ag/Ab and Hexagon		
	Result	Positive	Negative	Total
Determine (commercial buffer)	Positive	37	3	40
	Negative	0	96	96
Total		37	99	136

The sensitivity of the Determine HIV-1/2 test in this setting was 100% (95% CI 90.5 to 100), indicating that the RDT identified all individuals living with HIV, with a specificity of 97% (95% CI 91.4 to 99.4). The PPV was 92.5% (95% CI 79.6 to 98.4) and the test correctly identified 100% (95% CI 96.2 to 100) of those without the disease as not having the outcome of interest.

The agreement between the POCT and the serology test was 97.08%, with a κ of 0.93 (p<0.0001). Similarly, the Stuart–Maxwell test (df 2) showed no evidence (p=0.14) to reject the null hypothesis that the sensitivities of the two tests are equal.

## Discussion

HIV testing is essential for promoting individual well-being, ensuring access to healthcare and supporting public health initiatives in the fight against HIV/AIDS. It is a critical step for prevention and treatment and has a pivotal role in preventing mother-to-child transmission. RDTs are broadly used in LMICs as the first HIV test and are currently the Gambian Ministry of Health's standard of care.

Our study found that the Determine HIV-1/2 test used with PBS performed well in diagnosing HIV. Compared with the gold-standard algorithm, the sensitivity of the Determine HIV-1/2 was 100% (95% CI 90.5 to 100) and the specificity was 98% (95% CI 92.9 to 99.8). This met the WHO minimum requirements that an assay 1 test should have a sensitivity of at least 99%, a specificity of 98% and an invalid rate <5%.^3^ The CIs were wide, but even assuming a minimal sensitivity of 90.5%, the benefit of testing all individuals who require an HIV test would still outweigh the consequences of missing a diagnosis. In remote places, different barriers to accessing healthcare can be encountered, and losing the opportunity to test due to buffer scarcity might significantly impact the lives of PLHIV. Thus we consider it is better to test with a safe alternative solution and assume a 10% sensitivity loss than to not test when the opportunity arises. Individuals who tested positive are linked with an HIV service, undergo the diagnosis algorithm to confirm results, start early treatment to prevent advanced immunosuppression and transmission and receive counselling support. Those with negative results also receive information and are encouraged to retest and confirm their results with an approved WHO test to exclude a false negative result.

Although the test's specificity could be as low as 92.9%, the Determine HIV-1/2 with PBS can be used as a screening test when commercial buffer stockouts occur. Its results should always be confirmed by higher-specificity consecutive tests when these were available.

The positive likelihood ratio was high at 49.5 (95% CI 12.60 to 195), indicating that the test was good in establishing the diagnosis of HIV. The PPV was 94.9% (95% CI 82.7 to 99.4) and the NPV was 100% (95% CI 96.3 to 100). These, however, are prevalence dependent and should be cautiously interpreted in this cohort. Most participants were symptomatic at the time of testing. A much lower prevalence would be expected if the test were used in the general Gambian population, including those asymptomatic for screening purposes. In this scenario, false positives would increase, leading to a lower PPV. Further studies will be needed to evaluate the utility of PBS in asymptomatic individuals or in low-prevalence settings, where changes in estimates could result in high rates of misdiagnosis and waiting for the commercial buffer may be a better approach.

In addition, there was a high agreement between the Determine HIV-1/2 using PBS and the gold standard or the commercial kit. These findings were similarly reported by Mirambo et al.^29^ Nonetheless, it is essential to highlight that these parameters can vary with changes in prevalence. Moreover, the fact that two tests agree does not imply that the tests are correct. In our study design, this was overcome by making a three-way comparison.

There was insufficient evidence against the null hypothesis to conclude that the sensitivity and specificity between the Determine HIV-1/2 test using PBS and the manufacturer's buffer (p=0.66) or the gold-standard algorithm (p=0.22) differ. Yet these p-values should be cautiously interpreted, as the total number of discordant pairs was low and the test has low statistical power. Rejecting the null hypothesis cannot be interpreted as equivalence. For example, the test could have the same test accuracy but detect different populations.

Similar results were obtained when evaluating the performance of the Determine HIV-1/2 in the setting, and overall the test performed well in field conditions. Compared with the gold standard, the sensitivity was 100% (95% CI 90.05 to 100) with a specificity of 97% (95% CI 91.4 to 99.4). Test agreement with the gold-standard algorithm was good (κ=0.93) and invalid results were not recorded. These results are similar to others described in different LMICs.^30^

### Limitations

The sample size to achieve statistical power (0.9) was not met. The total number of recruited participants and performed tests was lower than expected. Regrettably, due to limitations in available resources, including personnel, time and funding, the study could not continue to achieve the desired sample size. When the decision was made to stop the study, results had not been analysed, but the study team was aware that the number of positives would meet the 80% power mark as defined by the sample size calculation.

Although most participants screened for enrolment agreed to participate in the study, only a limited number of tests were performed for screening purposes. Most individuals were symptomatic, indicating a more advanced stage of the disease. This was reflected by the fact that all the positive results with a fourth-generation test capable of detecting the infection in earlier stages were also positive with the POCT. As a result, the introduction of spectrum bias could have occurred, affecting the diagnostic accuracy and leading to a higher sensitivity than if the study had been conducted in the general population as a screening test. In such a population, individuals would be more likely to present with asymptomatic or earlier stages of the disease, increasing the probability of false negative results and decreasing the test's sensitivity. Moreover, some confounding medical conditions or different demographic groups could have been missed, introducing selection or spectrum bias. This could potentially affect the estimates if, for example, a condition that had been missed was a cause of cross-reactivity, with the RDT generating false negative or positive results.

Following the recommended WHO sequential testing approach can be arduous and time-consuming. While a single non-reactive test result is adequate to determine a definitive negative diagnosis, three positive assays are needed to confirm an HIV diagnosis. In our cohort, 18% of the results were not followed by a confirmatory test, which could introduce verification bias and lead to an overestimation of the sensitivity and specificity of the test. Fortunately, it only occurred on one occasion after a positive result, revealing that healthcare workers at the MRCG are generally aware of WHO testing algorithms and the importance of confirming positive results.

Perhaps this can be explained by the fact that the outpatient department is a busy clinic. Doctors see 20–30 patients daily, work under stress and simultaneously lead with different situations, which can increase the risk of medical errors, distraction and inattention, causing them to forget to request complementary tests. An alternative explanation is that some healthcare workers might not request confirmatory tests because they might be influenced by cultural beliefs or misinformation or feel distressed should they have to disclose a positive result after having had a negative one. In contrast, on the wards, patients are seen over a period by different personnel and missing results or complementary test requests are easier to detect.

On the other hand, half of the individuals that did not attend the phlebotomy room for serology confirmation did so despite their medical team having requested it, suggesting that although being persuaded to confirm their negative results, they were reluctant to retest. One possible explanation is the fear of having a positive result after a negative one and the impact this would have on their lives and care.

Furthermore, the generalisability of these results may be subject to limitations. PBS accuracy was only evaluated using a specific brand of rapid tests, and all tests were performed in a specific setting.

Further studies will be necessary to approach these limitations. Ideally, these should have larger sample sizes, include the general population and subjects at higher risk of infection and be carried out in different healthcare facilities, settings and LMICs.

Finally, errors during study performance could have occurred. Interpreting RDTs is subjective and each visual assay should be interpreted by at least two blinded readers with a variability rate of ≤5%.^21^ Unfortunately, due to logistic challenges in our study, the PBS RDTs were read by only one individual, which introduces the potential for misclassification bias or measurement errors.

### Strengths

Our study is the first to evaluate the sensitivity and specificity of the Determine HIV-1/2 test using an alternative buffer solution compared with a gold-standard algorithm. The study was designed in a three-way comparison and the agreement between the alternative buffer and the manufacturer was also evaluated.

Contrary to previous research^27–29^ where samples from blood banks were used, this study was performed in a prospective design with blind readers and under field conditions. All the index tests and the gold-standard samples were collected simultaneously, minimising the risk of discordant outcomes due to different infection window periods.

## Conclusions

Buffer stockouts and substitute liquid solutions are yet to be well studied. Ensuring all individuals requiring HIV RDTs can be tested and that correct buffer solutions are used when performing these is vital. The results of this study suggest that 0.01M PBS is an excellent alternative in the diagnosis of HIV using the Determine HIV-1/2 test. It is affordable, easy to prepare and feasible in most settings.

Using PBS could prevent potentially missed diagnoses when commercial testing kits are unavailable and would provide the opportunity to provide education and counselling to tested individuals regardless of their results. Nonetheless, this should always be followed by a WHO-prequalified test and recommended algorithms, and under no circumstances should results be interpreted as a definitive diagnosis. In addition, implementing policies to improve the minimum packaging requirement and training and educating healthcare workers to avoid buffer stockouts are vital and would be a definitive solution to this problem.

Further studies should evaluate the use of other buffer solutions (i.e. 0.9% saline) and the feasibility of using PBS in different settings. The risk–benefit relation of waiting for a commercial buffer versus utilising PBS for screening or in settings where the HIV prevalence is low should also be explored. In addition, future studies should evaluate interobserver variability and the utility of PBS in other fields, such as malaria and COVID-19, where buffer stockouts have also been described.

## Data Availability

Raw data is available upon request to the authors.
